# Association Between *GRIN1* rs28489906 and Major Depressive Disorder and Treatment Response to Antidepressants in Patients with Type 2 Diabetes

**DOI:** 10.3390/brainsci16030268

**Published:** 2026-02-27

**Authors:** Alejandra Monserrat Rodríguez-Ramírez, Mariela Benitez-Valenzuela, María Teresa Alcántara-Garcés, Marco Antonio Sanabrais-Jiménez, Julio Uriel Zaragoza-Hoyos, Humberto Del Valle-Ramírez, Viviana Hernandez-Romero, Augusto Rojas-Martínez, Ana Cristina García-Ulloa, Beatriz Camarena-Medellin

**Affiliations:** 1 Centro de Atención Integral del Paciente con Diabetes (CAIPaDi), Instituto Nacional de Ciencias Médicas y Nutrición Salvador Zubirán, Mexico City 14080, Mexico; alejandra.rodriguezr@incmnsz.mx (A.M.R.-R.); teresa.alcantarag@incmnsz.mx (M.T.A.-G.); humberto.delvaller@incmnsz.mx (H.D.V.-R.); ana.garciau@incmnsz.mx (A.C.G.-U.); 2 Escuela de Medicina y Ciencias de la Salud, Tecnologico de Monterrey, Monterrey 64710, Mexico; augusto.rojasmtz@tec.mx; 3 Departamento de Farmacogenética, Instituto Nacional de Psiquiatría Ramón de la Fuente Muñiz, Mexico City 14370, Mexico; masj@inprf.gob.mx (M.A.S.-J.); biojuzh@hotmail.com (J.U.Z.-H.); viviana_hdz@live.com (V.H.-R.)

**Keywords:** psychiatric genetics, affective disorders, antidepressants, diabetes

## Abstract

**Highlights:**

**What are the main findings?**

- The A allele of *GRIN1* rs28489906 was associated with MDD in T2D.
- The A allele was associated with decreased odds of treatment response.

**What are the implications of the main findings?**

- Potential identification of T2D patients at risk of MDD based on clinical characteristics and *GRIN1* genotype.
- This study contributes to the inclusion of genetics in antidepressant selection.

**Abstract:**

Introduction: Major depressive disorder (MDD) is a common comorbidity of type 2 diabetes (T2D) with low rates of treatment response to antidepressants. The pathophysiology of these conditions overlaps in N-methyl-d-aspartate receptors (NMDARs), which are present in neurons and pancreatic cells. *GRIN1* encodes NMDARs and has been associated with depression and T2D; however, its role in the coexistence of MDD and T2D and the treatment response to antidepressants has yet to be explored. Objective: We aimed to evaluate the association between *GRIN1* rs28489906 and MDD and treatment response to antidepressants in patients with T2D. Methods: A prospective study was conducted on T2D patients enrolled in a comprehensive care program with follow-up at 3 months. Subjects were assessed for MDD, and genotyping for *GRIN1* rs28489906 was performed. Depression scores and glycated hemoglobin (HbA1c) were analyzed using the Wilcoxon paired signed-rank test, and a stratified analysis was conducted to assess treatment response. Logistic regression analyses were performed to predict MDD and treatment response. Results: Our sample included 232 patients; among them, 49 (21%) had MDD. Patients with MDD showed a higher frequency of the AA genotype compared with non-MDD subjects (*p* = 0.026). A allele carriers reported lower treatment response rates (*p* = 0.049) and decreased odds of treatment response (OR = 0.03, 95% CI 0.002–0.63, *p* = 0.023). Patients with AG and AA genotypes with treatment response exhibited lower HbA1c (*p* = 0.029). Conclusions: The A allele of *GRIN1* rs28489906 was associated with MDD and treatment response in patients with T2D. Our findings highlight the role of glutamate in these comorbidities and the need for different therapeutic approaches based on genetics.

## 1. Introduction

Psychiatric disorders are frequent comorbidities in type 2 diabetes (T2D), among which major depressive disorder (MDD) is one of the most common conditions, affecting an estimated 14.5% of such patients [1,2]. This comorbidity has been associated with worse metabolic control, increased risk of diabetes-related complications, and increased mortality [3,4,5]. Improving depressive symptoms through the implementation of pharmacologic and non-pharmacologic interventions has been associated with an improvement in metabolic control in T2D [6]. Despite these findings, roughly 40% of patients with T2D and MDD do not report a treatment response to antidepressants [7], limiting available treatment options and exacerbating the impact of the condition. Multiple hypotheses have been explored to explain the higher prevalence of MDD in diabetes and the low rates of treatment response, including sedentarism, inadequate diet, and metabolic disturbances, among others. To address these challenges, in this study, we explore, through genetic analysis, a shared mechanism regarding the pathophysiology of these two conditions: N-methyl-d-aspartate receptors (NMDARs).

In recent studies, researchers have highlighted the role of glutamate in MDD, with strong evidence suggesting the involvement of glutamatergic pathways in the neurobiology of depression [8]. Glutamate is the main excitatory neurotransmitter in the central nervous system (CNS), and glutamatergic transmission largely mediates cognition and emotion. Evidence from postmortem histopathology and magnetic resonance imaging (MRI) studies demonstrated aberrant glutamatergic signaling in limbic and cortical regions of patients with MDD [9]. Glutamate binds to metabotropic and ionotropic receptors, such as N-methyl-d-aspartate receptors (NMDAR), to exert its functions [10]. These receptors are not only present in the CNS but also expressed in pancreatic cells, where NMDARs are involved in food intake regulation and energy balance [11], providing a link between the pathogenetic mechanisms of T2D and MDD. It can therefore be hypothesized that alterations in NMDAR may be a contributing factor in the high prevalence of MDD in these patients and their clinical characteristics, including their treatment response to antidepressants.

Genetics profoundly influences the expression and function of NMDAR. *GRIN1* is a gene that encodes the NR1 subunit of glutamate NMDAR, whose variants have been associated with neuropsychiatric disorders [12], TD2, diabetic retinopathy, and eating behavior alterations in diabetes [13]. In previous association studies, the authors have reported that genetic variation within *GRIN1* may contribute to susceptibility to the presence of depressive symptoms in clinical populations, supporting its relevance in affective phenotypes [14]. Moreover, variation in NMDA receptor genes, including *GRIN1*, has been associated with emotional and behavioral traits, reinforcing the biological plausibility of examining these variants in mood-related phenotypes [15]. Converging evidence indicates that glutamatergic dysfunction plays a key role in the neurobiology of MDD and represents an important therapeutic and pathophysiological target [16]. MDD frequently co-occurs with T2D, a comorbidity supported by shared neurobiological and metabolic mechanisms that may converge on glutamatergic pathways [17,18]. Within this framework, candidate polymorphisms in *GRIN1*, including intronic variants such as rs28489906, represent biologically plausible targets for genetic association studies aimed at understanding susceptibility to MDD in patients with metabolic comorbidities.

In light of the involvement of glutamatergic neurons in MDD and TD2 and the association of *GRIN1* with both conditions, investigation into this gene may contribute to a deeper understanding of these comorbidities and the search for treatment response biomarkers. We thus developed this study to evaluate the association between the *GRIN1* rs28489906 variant and MDD and treatment response to antidepressants in patients with T2D.

## 2. Materials and Methods

We conducted a prospective study with 3 months of follow-up in a multidisciplinary model of comprehensive care, the Center of Comprehensive Care for the Patient with Diabetes (CAIPaDi, an acronym for its name in Spanish) [19]. Our model includes evaluations and interventions from endocrinology, nutrition, ophthalmology, odontology, exercise, food care, diabetes education, psychology and psychiatry specialists. We performed two evaluations: baseline (enrollment in the center) and 3 months of treatment.

### 2.1. Study Population

Our study included Mexican patients over 18 years old who completed 3 months of treatment in an interdisciplinary clinic for diabetes between March and August of 2024. The included participants had been diagnosed with T2D for less than 5 years, had a body mass index (BMI) ≤ 45 kg/m^2^ without disabling complications, were non-smokers, and had no history of psychotic psychiatric disorders. Patients with substance use disorders were excluded. This study is registered on ClinicalTrials.gov (NCT02836808) and received approval from the Ethics and Research Committees of the National Institute of Medical Sciences and Nutrition Salvador Zubirán (Ref 1198). All participants were informed of the study purpose prior to inclusion and provided written consent. This study was conducted according to the standards of the Declaration of Helsinki.

Power calculations were performed using the “gap” package version 1.2.2 for RStudio version 2023.06.1+524. Assuming an additive genetic model, an allele frequency of 0.45, and a prevalence of 19% of MDD in patients with T2D, the analysis indicated a power of 0.93. Calculations were based on an alpha value of 0.05 and a proportion of 0.21 in a sample of 232 patients.

### 2.2. Procedure

We enrolled outpatients with T2D whose diagnosis was confirmed by means of blood test based on ADA (American Diabetes Association) diagnostic criteria (hemoglobin A1c ≥ 6.5% or fasting plasma glucose ≥ 126 mg/dL or random plasma glucose ≥ 200 mg/dL) during their first evaluation [20]. Patients were assessed at each appointment by all members of the interdisciplinary team [19] for T2D comprehensive care. An endocrinologist provided all pharmacologic interventions for metabolic control based on the ADA guidelines [21]. At baseline, we conducted a psychiatric clinical interview using DSM5 TR criteria for MDD diagnosis [22]. For additional standardization, a Mini International Neuropsychiatric Interview (M.I.N.I.) [23] was performed by a psychiatrist with all patients. We used the Hospital Anxiety and Depression Scale (HADS) subscale for depression [24] to assess symptom severity. All patients who were diagnosed with any psychiatric disorder, including MDD, were offered psychopharmacological treatment based on clinical practice guidelines [25]. Follow-up at 3 months also included a psychiatric evaluation and the use of HADS to assess symptom evolution. Consultation-liaison psychiatrists provided psychiatric evaluations and psychopharmacological treatments. Regardless of their acceptance of psychopharmacologic treatment, all patients received the standardized interventions of the program [19], which included exercise and nutritional advice, in addition to psychological monthly evaluations. Treatment response was defined as a reduction of >50% in the HADS depression score between baseline and follow-up evaluations [26] in the presence of antidepressant medication. Intervention response was defined as a reduction of >50% in the HADS depression score between baseline and follow-up in subjects without antidepressants.

The M.I.N.I. 5.0.0 Spanish version is a short diagnostic structured interview used to identify 16 psychiatric disorders based on the Diagnostic and Statistical Manual IV-TR edition (DSM-IV-TR) classification, including MDD [23,27]. The HADS is a self-administered tool composed of fourteen items and assesses psychological and somatic symptoms related to anxiety and depression [28]. Patients completed the HADS individually, and the scores were uploaded to our database by a member of our psychology team. We assigned groups at the baseline in agreement with the presence or absence of MDD.

Blood samples were taken during each medical appointment to determine fasting glucose, lipid profile (using colorimetric methods, SYNCHRON CX System (Beckman Coulter, Brea, CA, USA)), and HbA1c (using the HPLC method, Bio-Rad Variant II Turbo HbA1c Kit 2 (Bio-Rad Laboratories, Hercules, CA, USA)). Body composition was assessed by means of bioimpedance (body composition analyzer JAWON Medical IOI353 (JAWON Medical Co., Ltd., Gyeongsan, Republic of Korea)). An additional blood sample was taken at follow-up for the genetic analysis.

### 2.3. Genotyping

DNA was extracted from whole blood using the Flexigene DNA kit (Qiagen, Minneapolis, MN, USA). Genotyping of *GRIN1* rs28489906 was performed with the fluorogenic 5-exonuclease method using the C_1840191_10 TaqMan assay (Applied Biosystems, Foster City, CA, USA). The final volume was 7 µL with the following reaction conditions: 100 ng DNA, 2.5 µL TaqMan Master Mix, 2.367 µL water for PCR, and 0.125 µL 20x probe. The amplification and allelic discrimination were performed on StepOne real-time PCR systems (Applied Biosystems Inc., Foster City, CA, USA) with SDS v.2.1 software (Applied Biosystems).

### 2.4. Statistical Analysis

We utilized STATA 13.0 (StataCorp, 2013. Stata Statistical Software: Release 13. College Station, TX: StataCorp LP) and GraphPad Prism version 8.0 for Mac OS (GraphPad Software, La Jolla, CA, USA) for our statistical analyses. The normality of the variables was assessed using the skewness and kurtosis tests. Data are presented as means and standard deviations or medians and interquartile ranges for quantitative variables, depending on the distribution of each variable; categorical variables are presented as frequencies and percentages. The threshold for statistical significance in this study was set at *p* < 0.05. We used Student’s *t*-test to compare continuous variables with a normal distribution and the Mann–Whitney U test for variables with a non-normal distribution.

The association between categorical variables was evaluated using the χ^2^ test or the Fisher exact test, as appropriate. HADS scores and glycated hemoglobin (HbA1c) from baseline and follow-up were analyzed with the Wilcoxon paired signed rank test, and a stratified analysis was conducted to assess treatment responses by genotype due to the sample size and non-normal distribution of variables. For assessment of the association between MDD and *GRIN1*, we performed a logistic regression analysis using MDD as a categorical dependent variable and the *GRIN1* genotype as an independent variable; the model was subsequently adjusted to include sex and age as covariates. Another logistic regression model was established using treatment response as the dependent variable and *GRIN1* genotype as the independent variable; in addition, we included the variables that showed differences in the comparative analysis of responders and non-responders as independent variables (age and HbA1c). We used the Hosmer–Lemshow test to determine goodness of fit (*p* > 0.05).

## 3. Results

**Figure 1:**
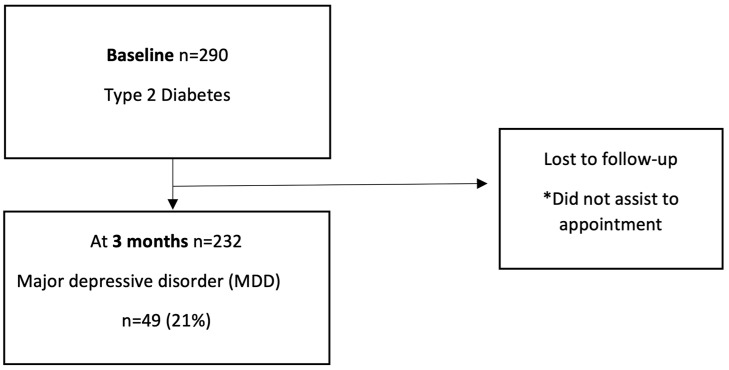
Flow chart of patient follow-up. * Cause of lost.

Our sample consisted of 232 subjects, the majority of whom were female, with a median age of 55 (46–62) years; among them, 49 (21%) met DSM-5 TR criteria for MDD and 183 (79%) had no depression (Figure 1).

No differences were recorded in sociodemographic variables and basal metabolic parameters between groups (Table 1). In the genetic analysis, we found significant differences between MDD and non-MDD groups; patients with MDD exhibited a higher frequency of the AA genotype compared with subjects without MDD (*p* = 0.026). Most patients with MDD carried the A risk allele of *GRIN1* rs28489906 (*p* = 0.049). In our logistic regression analysis, an association was found between MDD and the A risk allele of *GRIN1* rs28489906 (OR = 2.70, 95% CI 1.08–7.24, *p* = 0.047). We performed an analysis adjusted by age and sex, with the results showing no significant associations between the covariates or A risk allele of *GRIN1* rs28489906 and MDD (*GRIN1* rs28489906 OR = 2.41, 95% CI 0.89–6.52, *p* = 0.083; female sex OR = 1.73, 95% CI 0.88–3.40, *p* = 0.108; age OR = 0.97, 95% CI 0.93–1.008, *p* = 0.133).

Fifty participants (21%) from the entire sample underwent antidepressant treatment. Twenty-five non-MDD patients took antidepressant medication due to other clinical conditions such as anxiety disorders, eating disorders, fibromyalgia, and sleep disorders. Twenty-eight (57%) patients with MDD declined psychopharmacologic treatment; this group of subjects reported lower HADS depression scores at baseline (6 (3–9) vs. 9 (7–11), *p* = 0.009). Fluoxetine (median = 20 mg, IQR 20–20) was the most frequently prescribed antidepressant in the MDD and no MDD groups, followed by sertraline (median = 50 mg, IQR 50–75). Among MDD patients, only one participant was prescribed a medication other than SSRIs (duloxetine 60 mg). The non-MDD group more frequently reported the use of other types of antidepressants such as amitriptyline (median = 12.5 mg, IQR 6.75–25) or mirtazapine (7.5 mg). In the MDD group, 23 patients (47%) reported a reduction in depressive symptoms equivalent to treatment response (treatment/intervention response), with 10 (43%) of them taking an SSRI. The response rate to antidepressant medications was calculated at 40% in our sample. The majority of patients exhibiting treatment response were taking fluoxetine (median = 20 mg, IQR 20–20); no differences were found in the prescribed antidepressants between treatment and no-treatment response groups. Patients who presented a treatment response were younger than those exhibiting no response. Comparative analysis between MDD patients stratified by treatment or intervention response showed no significant differences in *GRIN1* rs28489906 genotypes (Table 2).

In agreement with our study design, we performed a Wilcoxon rank test of paired samples to assess the change in depressive symptoms between basal and follow-up evaluations by genotype in subjects with MDD. In the group taking SSRIs, we found that patients with the AA genotype exhibited no significant decrease in median HADS score between evaluations (7 (4–9) vs. 5 (2–7), *p* = 0.053); in comparison, patients with AG (9 (6–10.5) vs. 6 (3–7), *p* = 0.027) and GG (8.5 (7–10) vs. 4 (2–5), *p* = 0.044) genotypes exhibited reduced HADS depression scores at follow-up (Table 3).

**Figure 2:**
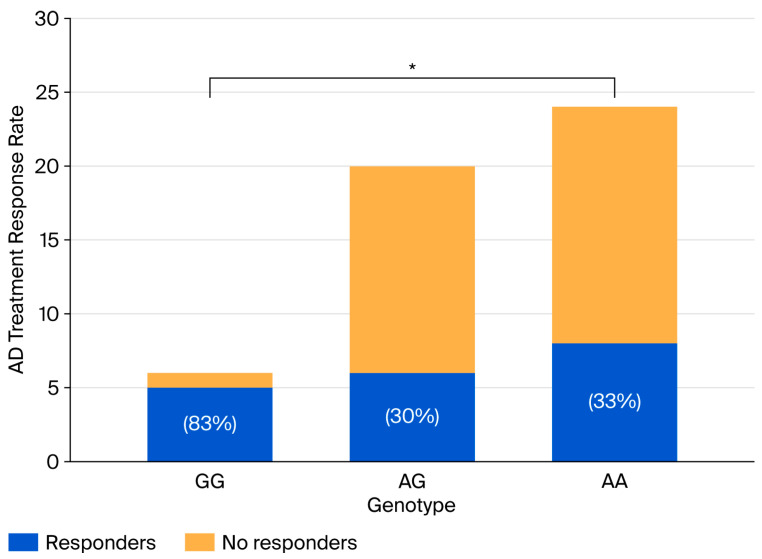
Antidepressant (AD) treatment response by *GRIN1* rs28489906 genotype. * *p* < 0.05.

Furthermore, patients with (Figure 2) AA and AG genotypes reported lower rates of response to antidepressant treatment compared with those with the GG genotype (9 (33%) vs. 5 (30%) vs. 4 (83%), *p* = 0.049). Patients not prescribed antidepressant medication reported significant reductions in HADS depression scores between evaluations in GG (4 (2–6) vs. 2 (1–4), *p* = 0.044) and AA genotypes (5 (3–8) vs. 3 (1–5), *p* = 0.006); in comparison, the score of those with AG genotypes remained unchanged (5 (3–8) vs. 3 (1–5), *p* = 0.118). Subjects with the GG genotype also reported higher response to the interventions (>50% of HADS depression score decrease in follow-up) rates compared with those with the AA and AG genotypes (25 (61%) vs. 27 (37%) vs. 30 (44%), *p* = 0.046).

We compared the metabolic parameters of MDD patients stratified by treatment response at follow-up, with our results revealing higher median HbA1c in those exhibiting no response (5.8% (5.6–6.2) vs. 6.4% (5.8–6.9), *p* = 0.042). No differences were found in the remaining metabolic parameters. We subsequently analyzed the HbA1c of MDD patients at baseline and follow-up by genotype. In terms of the three genotypes, significant reductions were reported in HbA1c in responders and non-responders (Table 4). In the group exhibiting treatment response, those with the GG genotype reported higher median HbA1c at follow-up; in comparison, at baseline and for both measures of the group without treatment response, there were no differences in HbA1c by genotype.

In agreement with our findings, we performed a logistic regression analysis in MDD patients to determine treatment response to antidepressants using A risk allele of *GRIN1* rs28489906, age, sex, and HbA1c at follow-up as independent variables. Our model (*p* = 0.0012, pseudo r^2^ = 0.267) showed that decreased odds of treatment response were associated with the A risk allele of *GRIN1* rs28489906 (OR = 0.03, 95% CI 0.0024–0.63, *p* = 0.023) and age (OR = 0.89, 95% CI 0.83–0.97, *p* = 0.008). Borderline results were recorded for HbA1c at follow-up (OR = 0.25, 95% CI 0.06–0.997, *p* = 0.050). In terms of sex, no significant association was recorded.

## 4. Discussion

In the present study, we analyzed the role of an SNP involved in glutamatergic pathways in depression and treatment responses to SSRI antidepressants in T2D patients. Our main findings include the association between *GRIN1* rs28489906 genotypes and MDD and depressive symptom reduction, regardless of antidepressant use, highlighting the role of the glutamatergic system in these comorbid conditions. As this is the first study to explore the genetic basis of a glutamate ionotropic receptor in this manner, several factors related to our findings must be discussed.

The prevalence of MDD in our group was higher than that reported in a recent meta-analysis [1], which may be in part conditioned by the composition of our sample, with more female participants and a younger age compared with other reports, both known risk factors for MDD in T2D [29,30,31]. It is also possible that the interdisciplinary approach of our center facilitated MDD detection and treatment.

In the genetics analysis, our results highlighted a higher frequency of the AG and AA genotypes of the *GRIN1* rs28489906 polymorphism in MDD patients, with a power of 0.93 based on our calculations, considering the MDD prevalence and sample size of this study. Through logistic regression analysis, we also found an association between the A allele of the *GRIN1* rs98489906 polymorphism and MDD, leading to the hypothesis that it is the risk allele for depression in TD2.

The *GRIN1* gene encodes the NR1 subunit of the NMDA receptor (N-methyl-D-aspartate), an essential component of the glutamatergic system involved in synaptic plasticity, learning, and memory [12,32]. The NR1 subunit, also known as GluN1, is involved in forming ionic channels for the influx of calcium, which leads to posterior neuronal depolarization [33,34]. The polymorphism rs28489906 is located in the second intron of the *GRIN1* gene; therefore, it does not alter protein composition. However, variations in these SNPs could alter transcription factor binding, generating a new protein-binding motif or modifying binding affinity, thereby affecting the binding of DNA transcription factors and modifying their regulatory function [35]. To assess whether the variant rs28489906 of *GRIN1* influences gene expression by altering transcription factor dynamics, we conducted a search using JASPAR [36] for DNA-binding motifs. We found that this polymorphism is located close to the DNA-binding motif of the ZNF189, TP53, and TP73 transcription factors. Together, ZNF189, TP53, and TP73 regulate transcriptional programs influencing cellular state and neuroplasticity via complementary mechanisms. ZNF189, a KRAB-C2H2 zinc finger, mediates TRIM28/KAP1-dependent epigenetic silencing and modulates stress-resilience circuitry [37]; TP53 converges genome-integrity and metabolic signals to coordinate repair, cell-cycle arrest, apoptosis, and senescence, consequently shaping inflammatory and metabolic signaling [38]; and TP73, despite partial target overlap with TP53, acts as a developmental regulator, preserving neurogenesis, hippocampal architecture, ependymal multiciliogenesis, and mitochondrial homeostasis [39]. We posit that the DNA-binding motif of ZNF189, TP53, and TP73, located near *GRIN1* rs28489906, may affect their interaction with transcription factors and their target genes, which impact NMDAR function.

Alterations in NMDAR have been previously associated with schizophrenia, bipolar disorder, major depressive disorder, anhedonia, and emotional dysregulation [12]. Despite the fact that information on the phenotype of *GRIN1* is incredibly limited, and no previous association between rs28489906 and MDD has been reported in the literature, research on other genes that encode the subunits of glutamatergic receptors has resulted in the identification of various polymorphisms related to depression and the clinical response to antidepressant treatment [12,32]. Lee LC et al. [15] examined the relationship between *GRIN1*, *GRIN2A*, *GRIN2B*, *GRIN2C*, and *GRIN2D* and emotions and social behavior in adolescents through the scores obtained in the Beck Youth Inventory. The authors reported an association between *GRIN1* rs4880213 and depression and disruptive behavior.

With regard to diabetes, NMDARs are also expressed on pancreatic cells, particularly in beta cells, where these receptors are involved in Ca^2+^ homeostasis, which mediates the stimulus–secretion cascades of pancreatic hormones such as insulin. NMDARs exert a positive regulatory effect on Ca^2+^ influx but also exert negative feedback through the activation of K^+^ channels [11,40]. Kochetova et al. [13] explored the role of four glutamatergic genes that code for ionotropic receptors of this neurotransmitter, including NMDAR, in T2D. In the above study, *GRIN2B* rs7301328, *GRINB2B* rs1805476, and *GRIA1* rs2195450 were associated with diabetes. Diabetic retinopathy was associated with *GRIK3* rs534131, *GRIA1* rs1805476, and *GRIN2B* rs7301328; in comparison, *GRIN1* rs6293 was associated with external eating behavior. Therefore, NMDAR dysfunction may lie at the core of the pathophysiological link between psychiatric comorbidities and T2D. Nonetheless, a greater number of studies are required to explore the presence of these *GRIN1* variants in other populations and explore their association with MDD in non-T2D subjects. Thus far, our findings on *GRIN1* suggest that MDD in T2D may form part of a phenotype characterized by NDMAR dysfunction preceded by a common *GRIN1* genotype.

Another objective of our study was to determine the association between *GRIN1* and treatment response; to achieve this aim, we first analyzed the clinical characteristics of our population. Our results revealed that 47% of patients with MDD reported a treatment response; however, only 43% of patients were taking an SSRI, with the remaining subjects declining treatment. Among the patients who initiated antidepressant therapy, 40% reported a treatment response. Our findings are in line with the extensive evidence on the limitations of SSRIs in the treatment of MDD and treatment response rates. In a recent reanalysis of the STAR*D trial [16,41], 41% of patients with MDD treated with a first-line SSRI responded to treatment; however, 70% did not achieve remission. In a study assessing T2D, Anderson et al. [7] reported a response rate of 59.9% in a group of patients taking sertraline or bupropion for MDD. One of the predictors of poor response was the use of sertraline, highlighting the limited effects of SSRIs in this population.

While analyzing the treatment response of our population, we observed an unpredicted finding. In our study, 56% of patients with MDD who reported improvement did not take antidepressants (intervention response). This phenomenon can be explained by the characteristics of our sample and the other interventions in our program. First, patients who declined antidepressant treatment reported a lower score of depression in the HADS baseline evaluation, making them more likely to exhibit improvement without medication. Furthermore, all patients participated in monthly psychological sessions and received exercise and nutritional counseling as part of the interdisciplinary program’s standard of care. For the treatment of mild and moderate depression, these non-pharmacologic interventions have proven efficacy in reducing depressive symptoms [26], which can explain the reduction in symptoms in the HADS at follow-up in the group without antidepressants. Lastly, the improvement in metabolic parameters at follow-up may also explain the improvement in depressive symptoms, as reported by previous studies [6,7,42].

The next stage in our study involved analyzing our genetic findings for treatment and intervention responses. The results of our logistic regression analyses revealed decreased odds of treatment response in MDD risk A allele carriers. It is important to note that A allele carriers in the group with depression reported more than a 95% reduction in treatment response odds. In the paired analyses, patients who carried two risk alleles (AA genotype) exhibited no significant change in the HADS depression score, even while taking antidepressants, and significant symptom reduction in the non-SSRI group. We contend that the severity of symptoms is also an important factor to take into consideration, given that patients without antidepressants reported lower HADS depression scores at baseline. Hence, patients with the AA genotype and mild symptoms of depression may report improvement with non-pharmacological approaches; in comparison, patients with the same genotype and moderate-to-severe depressive symptoms are less likely to respond to conventional SSRI treatment, even in combination with multidisciplinary interventions, suggesting the need for an antidepressant treatment with a different mechanism of action. Intriguingly, AG patients exhibited the opposite pattern of response, as non-antidepressant participants with less-severe depressive symptoms reported no improvement at follow-up, whereas patients with higher HADS scores and SSRI treatment reported a significant reduction in symptoms. Moreover, subjects without the risk allele (GG genotype) exhibited reduced HADS depression scores, irrespective of antidepressant use. Our findings are in line with those of previous studies suggesting that glutamatergic hyperactivity, or dysfunction in NMDA signaling, may be associated with reduced efficacy of conventional antidepressant treatment, which primarily acts on the monoaminergic system (serotonin and norepinephrine) but not directly on glutamate [16,43]. *GRIN1* rs28489906 has been seldom explored in MDD treatment response; research efforts have been directed toward other genes coding for NMDA receptor subunits. Chen et al. [44] reported that *GRIN2A* rs16966731 was associated with ketamine (a glutamatergic agent) treatment effects, and specific alleles of *GRIN2B* rs1805502 and rs890 [45,46] were associated with TRD in other studies. Our exploratory data on treatment response raise questions regarding the involvement of *GRIN1* in the effects of SSRIs and non-pharmacologic interventions, at least in T2D.

We analyzed the metabolic parameters of patients with MDD stratified by treatment response, observing a higher median HbA1c level at follow-up in those exhibiting no response to antidepressant treatment. Our results support the involvement of glycemic control in treatment response to antidepressants but also suggest that this response is influenced by *GRIN1* rs28489906 genotypes. In our sample, AG and AA subjects who exhibited treatment response reported lower HbA1c levels at follow-up than GG participants and the group that exhibited no treatment response. From these results, it is thus possible that patients who carry the A allele require lower HbA1c levels to achieve treatment response to antidepressants; in comparison, patients not harboring the risk allele may show improvements at higher HbA1c levels, thus prompting further questions.

As previously noted, the relationship between glycemic control and MDD is complicated and bidirectional. In one instance, depression can negatively affect treatment adherence, decrease motivation to maintain healthy habits [47], and alter the neuroendocrine mechanisms that regulate glucose metabolism [48]. As NMDARs are involved in the modulation of insulin secretion through the increase in extracellular and intracellular glutamate concentration, prolonged activation of these receptors, such as that mediated by the excitation of beta cells during states of chronic hyperglycemia, elevated serum concentrations of glutamate, and obesity-induced inflammation, could lead to the death of beta cells [11,49]. The results of a study conducted on mice showed that the deletion of the *GRIN1* gene from the NMDA receptor reduced glucose-induced insulin secretion and increased the death of beta cells under inflammatory conditions [11,50].

In comparison, an altered metabolic state can influence the antidepressant response and clinical course of MDD [7,51,52]. The results of previous studies have demonstrated a contribution of metabolic dysfunction to the activation of systemic inflammatory pathways, which can alter neuroplasticity and modify the sensitivity of the monoaminergic and glutamate receptors [11]. Additionally, in a study involving the use of proton magnetic resonance spectroscopy of the occipital cortex, the results showed an association between lower levels of glutamate and poor glycemic control in type 1 and 2 diabetes patients. Based on these findings, in T2D patients with poor glycemic control, it is possible to observe altered glutamatergic sensitivity co-existing with lower glutamate in the CNS. In carriers of the *GRIN1* rs28489906 A risk allele, NMDAR alterations may underlie the clinically observed low antidepressant treatment response in T2D and the need for lower HbA1c levels to achieve adequate response.

These findings support the hypothesis that the glutamatergic pathways could constitute a common therapeutic target for T2D, in addition to certain depressive disorders associated with metabolic dysfunction and inflammation. Further research is required to explore the treatment applications of the involvement of glutamatergic pathways in the coexistence of T2D and MDD.

This is the first study in which *GRIN1* rs28489906 is associated with MDD and treatment response to antidepressants. Despite the frequent co-occurrence of these conditions, research on their genetic basis in clinically complex cases remains scarce. The strengths of this study reside in an innovative application of genetics to investigate a common problem, in Latin America, where 34 million people live with T2D [53]. The population of our study is underrepresented in genetics studies and consortia, which primarily involve subjects of European ancestry and focus either on psychiatric or metabolic conditions, with little focus on the psychiatric genetics of subjects with both conditions from our region. The study of glutamatergic pathways in these patients provides an innovative perspective that contributes to a deeper understanding of these comorbid conditions. In addition, the longitudinal design of our study enabled us to observe clinical treatment response and enhance the accuracy of our data. As limitations, we acknowledge a lack of a non-T2D group to compare allele frequency and the limited sample size in the group with MDD, regardless of sufficient power calculations. Our analysis of treatment response to antidepressants must be replicated in larger sample sizes before clinical extrapolation.

## 5. Conclusions

In this study, we explored the genetic basis of MDD and treatment response to antidepressants in T2D through the analysis of a gene involved in glutamatergic neurotransmission. Our main findings include the association between *GRIN1* rs28489906 and MDD diagnosis and the identification of decreased odds of treatment response in A allele carriers. Additionally, we identified an association between glycated hemoglobin and treatment response to antidepressants that is central in A allele carriers. Based on our findings, we are compelled to highlight the need for further research on the genetic basis of glutamatergic receptors and their systemic interrelation to develop different therapeutic approaches based on genetics in these comorbidities.

## Figures and Tables

**Table 1:** Comparative analysis of clinical and genetic characteristics of patients stratified by MDD status.

Variable	MDD (n = 49)	No MDD (n = 183)	*p*
Age in years, median (IQR)	54 (46–61)	56 (46–62)	0.248
Female sex, n (%)	31 (63)	94 (51)	0.138
School in years, median (IQR)	12 (9–16)	12 (10–16)	0.354
Years of T2D diagnosis, median (IQR)	1 (0–3)	1 (0–3)	0.359
Eating disorder, n (%)	9 (18)	19 (10)	0.128
HADS depression score, median (IQR)	8 (5–10)	5 (3–8)	0.000
Antidepressants, n (%)	25 (51)	25 (13)	0.000
- Fluoxetine, n (%)	15 (31)	12 (6)	0.000
- Sertraline, n (%)	4 (8)	6 (3)	0.225
- Citalopram, n (%)	2 (4)	0	0.044
- Escitalopram, n (%)	3 (6)	1 (0.5)	0.030
- Paroxetine, n (%)	0	1 (0.5)	1.000
- Duloxetine, n (%)	1 (2)	1 (0.5)	0.379
- Amitriptiline, n(%)	0	2 (1)	1.000
- Mirtazapine, n (%)	0	2 (1)	1.000
*GRIN1* rs28489906, n (%)			
GG	5 (0.10)	43 (0.23)	0.026
AG	17 (0.35)	75 (0.41)	
AA	27 (0.55)	65 (0.36)	
Allele A *GRIN1* rs28489906, n (%)			
A allele carriers	44 (0.90)	140 (0.76)	0.049
No A allele carriers	5 (0.10)	43 (0.24)	
**Baseline metabolic parameters**			
Fasting glucose (mg/dL), median (IQR)	130.5 (110–195)	132 (108–178)	0.850
HbA1c %, median (IQR)	8.2 (6.75–9.9)	7.5 (6.4–9.6)	0.174
Total cholesterol (mg/dL), median (IQR)	181 (154–204)	185 (160–212)	0.554
LDL cholesterol (mg/dL), median (IQR)	104 (89–129)	111 (91–138)	0.273
HDL cholesterol (mg/dL), median (IQR)	40 (35–48)	41 (35–48)	0.661
Non-HDL cholesterol (mg/dL), median (IQR)	144 (112–156)	144 (116–165)	0.562
Triglyceride (mg/dL), median (IQR)	179 (142–259)	162 (119–235)	0.110
Body mass index (kg/m^2^), median (IQR)	28 (26.3–31)	29.1 (25.8–32)	0.863
**Follow-up metabolic parameters**			
Fasting glucose (mg/dL), median (IQR)	98.5 (92–115)	100 (90–111)	0.743
HbA1c %, median (IQR)	6.1 (5.7–6.7)	6.2 (5.9–6.6)	0.537
Total cholesterol (mg/dL), median (IQR)	153 (130–175)	151 (131–173)	0.896
LDL cholesterol (mg/dL), median (IQR)	88 (74–98)	86 (73–104)	0.693
HDL cholesterol (mg/dL), median (IQR)	41 (37–49)	44 (38–52)	0.409
Non-HDL cholesterol (mg/dL), median (IQR)	105 (94–123)	107 (87–122)	0.789
Triglyceride (mg/dL), median (IQR)	138 (95–165)	117 (94–156)	0.490
Body mass index (kg/m^2^), median (IQR)	27.6 (25.6–31.6)	28.1 (25.3–30.8)	0.947

Major depressive disorder (MDD); Hospital Anxiety and Depression Scale (HADS).

**Table 2:** Comparative analysis of genotype and metabolic parameters at follow-up by treatment/intervention response.

Variable	MDD Treatment/Intervention Response (n = 23)	MDD No Treatment/Intervention Response (n = 26)	*p*	No MDD n = 183	*p*
Female sex, n (%)	12 (52)	19 (73)	0.151	94 (51)	0.903
Age (years), median (IQR)	49 (45–60)	59.5 (53–64)	0.006	56 (46–62)	0.0101
Years of T2D diagnosis, median (IQR)	1 (0–3)	0.5 (0–1)	0.228	1 (0–3)	0.3559
Antidepressant use, n (%)	10 (43)	15 (57)	0.321	25 (13)	<0.001
- Fluoxetine, n (%)	8 (35)	7 (27)	0.757	12 (6)	<0.001
- Sertraline, n (%)	1 (4)	3 (11)	0.612	6 (3)	0.119
- Citalopram, n (%)	0	2 (8)	0.491	0	0.022
- Escitalopram, n (%)	1 (4)	2 (8)	1.000	1 (0.5)	0.030
- Paroxetine, n (%)	0	0	-	1 (0.5)	1.000
- Duloxetine, n (%)	0	1 (4)	1.000	1 (0.5)	0.379
- Amitriptiline, n(%)	0	0	-	2 (1)	1.000
- Mirtazapine, n (%)	0	0	-	2 (1)	1.000
Fasting glucose (mg/dL), median (IQR)	100 (91–120)	107 (95–122)	0.336	100 (90–111)	0.154
HbA1c %, median (IQR)	5.8 (5.6–6.2)	6.4 (5.8–6.9)	0.042	6.2 (5.9–6.6)	0.287
Total cholesterol (mg/dL), median (IQR)	155 (138–187)	160 (130–175)	0.696	151 (131–173)	0.849
LDL cholesterol (mg/dL), median (IQR)	92 (75–107)	88 (76–91)	0.434	86 (73–104)	0.925
HDL cholesterol (mg/dL), median (IQR)	42 (38–48)	42 (36–49)	0.725	44 (38–52)	0.694
Non-HDL cholesterol (mg/dL), median (IQR)	113 (95–128)	110 (90–128)	0.602	107 (87–122)	0.896
Triglyceride (mg/dL), median (IQR)	119 (89–168)	145 (100–176)	0.214	117 (94–156)	0.287
Body mass index (kg/m^2^), median (IQR)	28 (25.4–29.6)	27.4 (25.8–31.6)	0.846	28.1 (25.3–30.8)	0.961
*GRIN1* rs28489906, n (%)					
GG	4 (0.17)	1 (0.4)		43 (0.23)	0.060
AG	8 (0.35)	9 (0.35)		75 (0.41)	
AA	11 (0.48)	16 (0.61)	0.262	65 (0.36)	
A allele carriers, n (%)	19 (0.83)	25 (0.96)	0.173	140 (0.76)	0.054
No A allele carriers, (%)	4 (0.17)	1 (0.4)		43 (0.24)	

Major depressive disorder (MDD).

**Table 3:** Comparative analysis of HADS depression scores at baseline and follow-up by *GRIN1* rs28489906 genotypes in patients with MDD stratified by antidepressant use.

	*GRIN1* rs28489906	HADS-d Score Baseline	HADS-d Score Follow-Up	*p*
**MDD No AD**	GG	4 (2–6)	2 (1–4)	0.002
	AG	5 (3–8)	4 (2–5)	0.118
	AA	5 (3–8)	3 (1–5)	0.006
	***p***	0.450	0.006	
**MDD AD**	GG	8.5 (7–10)	4 (2–5)	0.044
	AG	9 (6–10.5)	6 (3–7)	0.027
	AA	7 (4–9)	5 (2–7)	0.053
	***p***	0.402	0.616	

Major depressive disorder (MDD); Antidepressant (AD); Hospital Anxiety and Depression Scale depression score (HADS-d).

**Table 4:** Comparative analysis of HbA1c at baseline and follow-up by *GRIN1* rs28489906 genotypes in patients with MDD stratified by treatment response.

	*GRIN1* rs28489906	HbA1c Baseline	HbA1c Follow-Up	*p*
**MDD treatment response**	GG	8.67 (7.1–10.25)	6.55 (6.25–7.2)	0.0000
	AG	8 (6.25–9.45)	5.6 (5.4–6.05)	0.0002
	AA	8.9 (7.5–9.5)	5.9 (5.7–6.8)	0.0000
	***p***	0.401	0.029	
**MDD No treatment response**	GG	7.3	6.9	0.0005
	AG	9.07 (6.9–10.9)	6.4 (5.8–6.6)	0.0000
	AA	8.1 (6.25–9.75)	6.4 (5.95–6.95)	0.0000
	***p***	0.704	0.521	

Major depressive disorder (MDD); Glycated hemoglobin (HbA1c).

## Data Availability

The data presented in this study are available from the corresponding author upon request due to institutional policies for the protection of patients’ privacy.

## Abbreviations

The following abbreviations are used in this manuscript:

SSRI	Selective serotonin reuptake inhibitor
T2D	Type 2 diabetes
MDD	Major depressive disorder
NMDARs	N-methyl-d-aspartate receptors
